# Local arrangement of microfibrillar-associated protein 5 with neurovascular and extracellular components in non- and ischemia-affected brain regions of mice

**DOI:** 10.3389/fnins.2025.1593948

**Published:** 2025-06-06

**Authors:** Corinna Höfling, Steffen Roßner, Bianca Flachmeyer, Wolfgang Härtig, Dominik Michalski

**Affiliations:** ^1^Department of Neurology, University of Leipzig, Leipzig, Germany; ^2^Paul Flechsig Institute—Centre of Neuropathology and Brain Research, University of Leipzig, Leipzig, Germany; ^3^Institute of Anatomy, University of Leipzig, Leipzig, Germany

**Keywords:** stroke, focal cerebral ischemia, neurovascular unit, NVU, microfibrillar-associated protein 5, MFAP5, NF-L, MAP2

## Abstract

Stroke often leads to death or functional impairment, and neuroprotective strategies are still lacking. Among the mechanisms contributing to tissue damage and yielding thus interest for therapeutic interventions, an affection of cytoskeletal elements has been considered. A first description of the microfibrillar-associated protein 5 (MFAP5) has yielded a fiber-like pattern and reduced immunosignals in the ischemic brain. However, details on region characteristics are lacking. This study thus aimed to explore local arrangements of MFAP5 with components of the neurovascular unit and extracellular matrix in non- and ischemia-affected neocortical brain regions of mice. Immunofluorescence labeling was used to visualize MFAP5 simultaneously with neurons, glial cells, vasculature, perineuronal nets, fibronectin, and the cytoskeletal elements neurofilament light chain (NF-L) and microtubule-associated protein 2 (MAP2). Fluorescence-based microscopy, confocal laser scanning microscopy, and 3D surface reconstruction served for analyses. MFAP5 was observed in a predominantly fiber-like and partially surrounding formation associated with neuronal processes and cell bodies. In the ischemic region, MFAP5 markedly diminished, but a few fiber-like structures were maintained with a thinned, partially fragmented, and twisted aspect. MFAP5 exhibited no clear regional association with microglia, astroglia, or parts of the vasculature and ECM. However, the local arrangement of MFAP5 and its change due to ischemia was comparable to that of NF-L and MAP2. This study comprehensively described MFAP5 after experimental stroke and identified similarities with MAP2 and NF-L. Thus, MFAP5 might represent an essential component of the neuronal cytoskeleton. Further research is needed to explore its functional properties and potential for neuroprotective approaches.

## 1 Introduction

Ischemic stroke is a significant cause of death and functional impairment impacting the individual health-related quality of life in survivors (Martin et al., 2024; Tengs et al., 2001). Causal therapies currently focus on re-opening occluded vessels and thus restoring sufficient blood supply to brain tissue. Among these approaches, intravenous thrombolysis and endovascular treatment have proven beneficial regarding mortality and the degree of functional impairment (Hilkens et al., 2024). However, many patients are treated without a relevant effect or are not eligible for these therapies for reasons such as late hospital arrival (Dhillon et al., 2023; Hilkens et al., 2024).

Neuroprotective strategies are thus needed and might be applied alone or in combination with recanalizing approaches in the early stage of stroke (Eren and Yilmaz, 2022; Zhang et al., in press). A detailed understanding of mechanisms underlying ischemic tissue damage is essential in this light. On the cellular level, several mechanisms, such as excitotoxicity, edema formation, and secondary inflammation, have been identified (Dirnagl et al., 1999). Regarding the type of brain cells involved in tissue damage, the concept of the neurovascular unit (NVU) has been established, describing the regional composition of neurons, glial cells such as astrocytes and oligodendrocytes, and the vasculature (del Zoppo, 2010; Schaeffer and Iadecola, 2021). Meanwhile, neuroprotective considerations in the setting of stroke widely include the NVU concept and particularly the different vulnerabilities of its neuronal, vascular, and glial components over time (Lyden et al., 2021). In addition to cellular structures, the extracellular matrix (ECM) is typically affected by ischemia, while alterations of its polyanionic perineuronal nets seem to occur concomitantly with those of NVU components (Härtig et al., 2017; Härtig et al., 2022). Modifications of the NVU and ECM are accompanied by various damaging and protective processes simultaneously, not only in acute but also later and, thus, regenerative phases of ischemia (Endres et al., 2008). Therefore, the emerging insights into pathophysiological processes have led to more complex neuroprotective approaches considering the temporal evolution of stroke (Zhao et al., 2024).

However, knowledge is still limited regarding the transition from reversible to irreversible tissue damage due to focal cerebral ischemia. Among others, cell-stabilizing, particularly cytoskeletal elements, have been discussed to critically impact cellular integrity in the setting of ischemia (Dewar and Dawson, 1997; Yuan et al., 2012). This perspective is supported by studies showing significant alterations of, for instance, neurofilament light chain (NF-L) and microtubule-associated protein 2 (MAP2) due to experimental stroke (e.g., Härtig et al., 2016; Mages et al., 2018; Mages et al., 2021). The importance of cytoskeletal elements is further strengthened by studies that have shown a correlation of NF-L and MAP2 serum levels with the size of infarction and the clinical outcome in stroke patients (e.g., Holmegaard et al., 2024; Uphaus et al., 2019; Mages et al., 2021; Barba et al., 2025). Thus, cytoskeletal elements, i.e., NF-L and others, might serve as biomarkers in neurological disorders, including stroke, to assess tissue damage and allow prognostication more precisely (Sharma et al., 2024).

As a further group of elements that may significantly contribute to cellular stabilization, microfibrillar-associated proteins (MFAPs) have been considered. MFAPs represent a family of extracellular matrix glycoproteins believed to be involved in the assembling of elastic microfibrils and inflammatory processes, which was investigated in obesity and cancer diseases (e.g., Vaittinen et al., 2015; Zhu et al., 2021) and in the scar formation of the skin (Han et al., 2023). In the first approach of visualizing MFAPs in the brains of mice suffering from experimental stroke, MFAP5 was detected with a fiber-like pattern, and respective immunosignals were found to be significantly reduced in ischemic areas along with that of NF-L and MAP2 (Höfling et al., 2023).

However, details on regional characteristics of MFAP5 concerning neurons, glial cells, vasculature, extracellular components, and cell-stabilizing elements are still lacking. Such information might help to get insight into the localization of MFAP5 and thus pave the way for further research addressing its functional properties and the potential for neuroprotective approaches. Therefore, this study aimed to explore local arrangements of MFAP5 with components of the NVU, ECM, and cytoskeleton in non- and ischemia-affected brain regions.

## 2 Methods

### 2.1 Study design

This explorative study was based on brain tissues from 6 male C57BL/6J mice suffering from experimental focal cerebral ischemia for 24 h. Mice were obtained from Charles River Laboratories (Sulzfeld, Germany) and had a mean body weight of 25 g. Multiple immunofluorescence labelings were used to visualize MFAP5 in non- and ischemia-affected brain regions together with various components of the NVU and ECM as well as cell-stabilizing elements (overview in Table 1). Fluorescence-based microscopy, confocal laser scanning microscopy, and 3D surface reconstruction were used for qualitative analyses.

**TABLE 1:** List of applied immunoreagents.

Primary antibodies/lectins	Abbr.	Dilution	Order number	Company	Secondary antibodies/streptavidin conjugates
Microfibrillar-associated protein 5	MFAP5	1:100	HPA010553-2	Merck (Sigma-Aldrich)	Donkey anti-rabbit Cy3
Neuronal nuclei	NeuN	1:1,000	MAB377	Merck (Millipore)	Donkey anti-mouse Cy5
Human C and D protein	HuC/D	1:1,000	A-21271	Thermo Fisher (Invitrogen)	Donkey anti-mouse Cy5
Myelin basic protein	MBP	1:100	ab7349	Abcam	Donkey anti-rat Cy2
Ionized calcium-binding adapter molecule 1	Iba1	1:200	234004	Synaptic Systems	Donkey anti-guinea pig Cy5
Aquaporin 4	Aqp4	1:500	sc-9888	Santa Cruz	Donkey anti-goat Cy2
Glial fibrillary acidic protein	GFAP	1:100	G6171	Merck (Sigma-Aldrich)	Donkey anti-mouse Cy5
β-actin		1:50	A5316	Merck (Sigma-Aldrich)	Donkey anti-mouse Cy2
Neurofilament light chain	NF-L	1:50	171014	Synaptic Systems	Donkey anti-guinea pig Cy5
β3-tubulin		1:100	302304	Synaptic Systems	Donkey anti-guinea pig Cy2
Microtubule-associated protein 2	MAP2	1:100	188011	Synaptic Systems	Donkey anti-mouse Cy5
*Solanum tuberosum* lectin, biotinylated	STL	1:500	B-1165	VectorLabs	Streptavidin Cy2
Collagen IV	Coll IV	1:1,000	AB769	Merck (Sigma-Aldrich)	Donkey anti-goat Cy5
*Wisteria floribunda* lectin, biotinylated	WFA	1:100	B-1355	VectorLabs	Streptavidin Cy2
Fibronectin	FN	1:100	IMS02-060-314	Agrisera	Donkey anti-chicken Cy5

Animal experiments were performed in accordance with the European Union Directive 2010/63/EU and the German guidelines for the care and use of laboratory animals. Reporting followed the ARRIVE criteria (Percie du Sert et al., 2020). Experiments were approved by the Landesdirektion Sachsen (Leipzig, Germany) as the local authority (reference number TVV 02/17).

### 2.2 Experimental focal cerebral ischemia

Focal cerebral ischemia was induced by an intraluminal, i.e., filament-based model, which is intended to critically reduce blood supply in the territory of the middle cerebral artery. It combines advantages such as the absence of craniectomy (Mergenthaler and Meisel, 2012). The applied model was described by Longa et al. (1989) and used with some minor modifications. In brief, the right-sided cervical arteries were prepared using a microscope (Carl Zeiss Microscopy, Oberkochen, Germany), and a highly standardized, silicon-coated 6-0 filament (Doccol Corporation, Redlands, CA, United States) was inserted and moved forward in the internal carotid artery until the origin of the middle cerebral artery. From that moment, the filament was left in place. During surgery, deep anesthesia was ensured with 2–2.5% isoflurane (Baxter, Unterschleißheim, Germany) in a mixture of 70% N_2_O/30% O_2_ using a vaporisator (VIP 3000, Matrix, New York, United States). To avoid anesthesia-related cooling, the mice’s body temperature was monitored and adjusted to 37°C with a thermostatically regulated warming pad (Fine Science Tools, Heidelberg, Germany). Animals received a complex pain medication with local and systemic components.

To assess neurobehavioral deficits, a score by Menzies et al. (1992) with a range from 0 (no observable deficits) to 4 (spontaneous contralateral circling) was determined during the 24-h period after surgery. Thereby, sufficient induction of focal cerebral ischemia was presumed if mice presented a score of at least 2, which was achieved by all mice included in the study (mean Menzies score of 3.4).

### 2.3 Tissue preparation and multiple fluorescence labeling

Brain tissues originated from mice transcardially perfused with 0.9% saline solution and 4% phosphate-buffered paraformaldehyde (PFA). Fixed brains were equilibrated with 30% phosphate-buffered sucrose and cut with a freezing microtome (Leica SM 2000R, Leica Biosystems, Wetzlar, Germany). Sections with 30 μm thickness were collected in 0.1 M phosphate buffer containing 0.025% sodium azide until histochemical stainings.

All incubations described below were separated by three washing steps with Tris-buffered saline (TBS; 0.1 M, pH 7.4) for at least 5 min. Antigen retrieval was performed in 60% methanol for 60 min. Afterward, sections were incubated for 30 min with 0.3% Triton X-100 in TBS supplemented with normal donkey serum (Dianova, Hamburg, Germany) and in case of primary antibodies originating from mice, additional donkey anti-mouse Fab fragments (Dianova, Hamburg, Germany). Primary antibodies and lectins were added in a mixture of 0.1% Triton X-100 and normal donkey serum in TBS, and free-floating slices were incubated overnight at room temperature. The following day, carbocyanine (Cy)-conjugated secondary antibodies and streptavidin containing 2% bovine serum albumin were applied to TBS. After incubation for 1 h, extensively rinsed sections were mounted onto glass slides and coverslipped.

### 2.4 Microscopy and analyses of fluorescence signals

Brain sections, stained with Cy-labeled immunoreagents, were scanned with an AxioScan.Z1 microscope (Carl Zeiss, Oberkochen, Germany) equipped with a Colibri7 light source. Images of brain slices were taken by the Axiocam 506 and a Plan-Apochromat objective (20x/0.8; Carl Zeiss) with an exposure time of 7 ms for Cy2, 10 ms for Cy3, and 5 ms for Cy5. Images were digitized using the ZEN 2.6 software (Carl Zeiss).

The upright confocal laser-scanning microscope LSM 880 (Carl Zeiss) was used for imaging of immunohistochemically stained samples in high-resolution mode by using an additional airyscan detector. The fluorescence was excited with an argon laser (488 nm, Cy2) and 2 HeNe lasers (563 nm, Cy3; 633 nm, Cy5). The emitted light was collected using three band-pass filters: 505–530 nm for Cy2, 565–615 nm for Cy3, and 650–710 nm for Cy5. The images were taken with a 63x immersion C-Apochromat objective (1.2 water DIC) and a zoom of 2. Images for 3D reconstruction were taken with a zoom factor of 4 and a z-stack with a 0.21-0.27 μm interval in an average range of 5 μm.

Image digitalization and processing for 3D surface reconstruction was performed with ZEN black 3.0 (Carl Zeiss) with the following applications: threshold of include signals was set to 50% for all channels by histogram thresholds of 500-5,000 for Cy2 and Cy3 and 500-4,000 for Cy5. The ambient for the iso-surface reconstruction was set to 0%, and shininess and light were set to 100%.

## 3 Results

**FIGURE 1:**
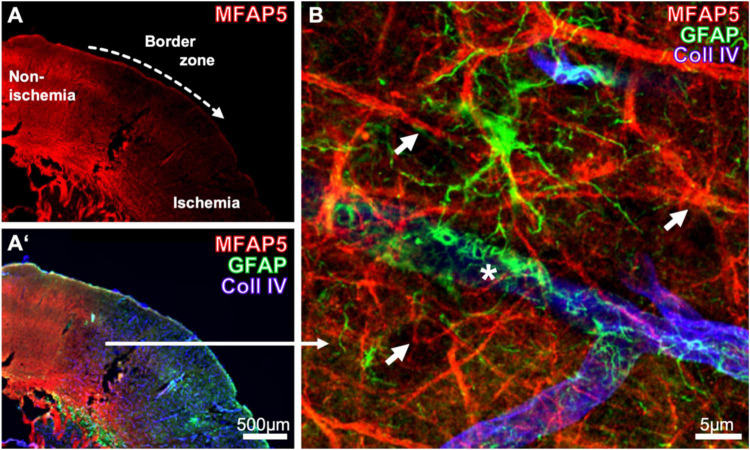
Immunohistochemical detection of microfibrillar-associated protein 5 (MFAP5) together with astroglial and vascular structures (glial fibrillary acidic protein, GFAP; collagen IV, Coll IV) in a representative mouse brain affected by 24 h of focal cerebral ischemia. **(A)** MFAP5 signal in the border zone between the non- and ischemia-affected neocortical brain regions. Simultaneous visualization of MFAP5, GFAP, and Coll IV in the neocortical border zone of ischemia **(B)**. Higher magnification of immunosignals originating from MFAP5, GFAP, and Coll IV at the ischemic border zone **(C)**. Scale bars are labeled in related figures, while the scale bar of **(A’)** is also valid for **(A)**. Meanings of asterisk and arrows are described in the main text.

Neocortical brain regions affected by focal cerebral ischemia were recognized by altered immunosignals of MFAP5 and components of the NVU. Overall, the MFAP5 signal appeared lesser toward the ischemic region (Figure 1A), accompanied by an enhanced visualization of the collagen IV and an opposite glial fibrillary acidic protein (GFAP) signal, indicating affected vascular and astroglial structures (Figure 1A’). In the ischemic border zone, higher magnification revealed MFAP5 predominantly as fibers with different diameters (arrows in Figure 1B). These were diffusely arranged without a clear regional association to the exemplarily visualized vascular and astroglial components of the NVU, while the latter were partially found in close vicinity (asterisk in Figure 1B).

Subsequent analyses were focused on MFAP5 in non- and ischemia-affected neocortical regions. While the non-ischemic hemisphere was used for comparison, the border zone and the mainly affected region were considered on the ischemic hemisphere. Thereby, local arrangements of MFAP5 were assessed concerning neurons, glial and vascular components of the NVU, the ECM, and cytoskeletal elements.

### 3.1 Regional characteristics of MFAP5, neurons and glial structures

**FIGURE 2:**
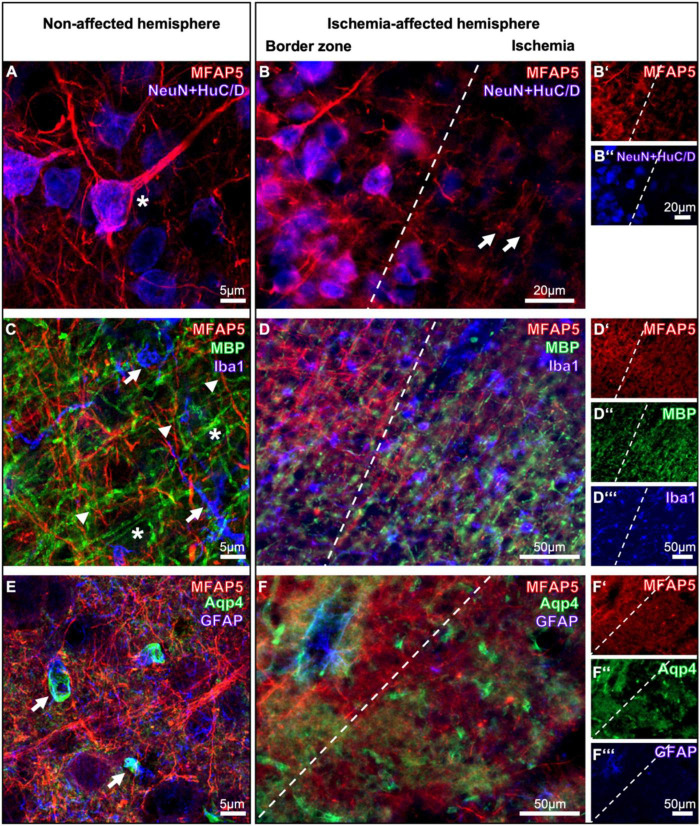
Multiple immunofluorescence labeling of the microfibrillar-associated protein 5 (MFAP5) together with representative structures from neurons (Neuronal Nuclei, NeuN; human protein C and d, HuC/D), oligodendrocytes (myelin basic protein, MBP), microglia (ionized calcium-binding adapter molecule 1, Iba1), and astroglia (aquaporin 4, Aqp4; glial fibrillary acidic protein, GFAP) in neocortical brain regions in mice, affected by focal cerebral ischemia for 24 h. For each combination of markers, the local arrangement is compared between the non-affected **(A,C,E)** and the ischemia-affected hemisphere **(B-B”,D-D”’,F-F”’)**. Scale bars are labeled in related figures, while the scale bar in **(B”)** is also valid for **(B’)**, and the scale bars in **(D”’,F”’)** are also valid for **(D’,D”,F’,F”)**. Meanings of asterisk, arrows, and arrowheads are described in the main text.

Neuronal nuclei (NeuN) and the human C and D protein (HuC/D) staining was simultaneously applied to visualize neurons. In a combined staining approach, MFAP5 appeared in a fiber-like and partially surrounding formation associated with neuronal processes and cell bodies in the non-affected hemisphere (asterisk in Figure 2A). This kind of local arrangement was observed comparably in the ischemic bordering zone, whereby the ischemic region exhibited diminished immunosignals of both neurons and MFAP5. Remarkably, neurons appeared almost completely lost in the ischemic neocortex, while a weak signal of MFAP5 remained that indicated thinned and partially fragmented fiber structures (arrows in Figure 2B).

As components of the NVU’s glial part, oligodendrocytes were visualized by the myelin basic protein (MBP), microglia by the ionized calcium-binding adapter molecule 1 (Iba1), and astroglia by aquaporin 4 and the glial fibrillary acidic protein (GFAP) (Figures 2C-F). In the non-ischemic hemisphere, the diffusely arranged MFAP5-positive fibers did not show a clear regional association with typically ramified microglia (arrows in Figure 2C) and myelin sheaths related to oligodendrocytes (asterisks in Figure 2C). While the fiber-like signals of MFAP5 and oligodendrocytic structures seemed to cross each other, they did not match in a joint cellular constituent (arrowheads Figure 2C). On the ischemia-affected hemisphere, the same pattern was observed in the border zone and the ischemic region. The latter exhibited a slightly enhanced oligodendrocytic signal and a reduced immunoreactivity for MFAP5 and morphologically altered microglia (Figure 2D).

Moreover, MFAP5 did not show a regional association with the astroglial GFAP. In contrast, GFAP and aquaporin 4 partially overlapped close to the vasculature, probably representing astrocytic endfeet (arrows in Figure 2E). Under ischemic conditions, the immunosignals of MFAP5 appeared lesser concomitantly to those originating from astrocytic structures. Comparable to the situation in non-affected regions, MFAP5-positive fibers that remained under ischemic conditions did not show a close regional association with astrocytic fragments (Figure 2F).

### 3.2 Association of MFAP5 with the vasculature and constituents of the ECM

**FIGURE 3:**
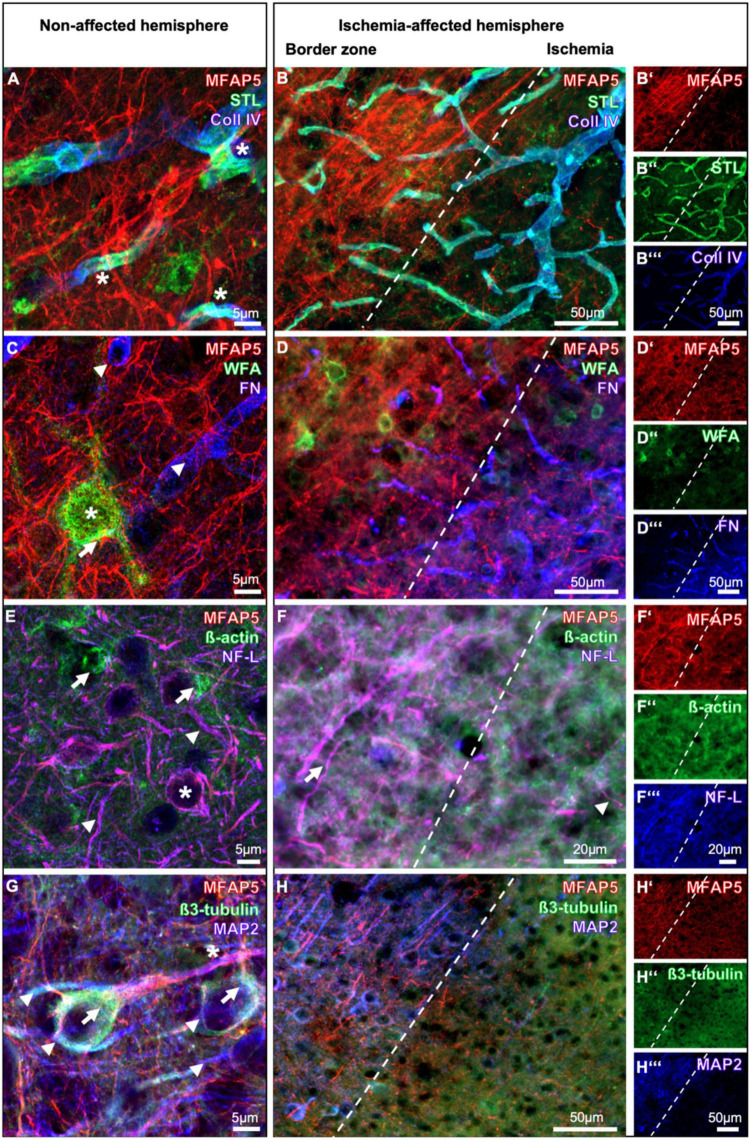
Immunofluorescence-based visualization of the microfibrillar-associated protein 5 (MFAP5) combined with the detection of vascular elements (*Solanum tuberosum* lectin, STL; collagen IV, Coll IV), perineuronal nets of the extracellular matrix (*Wisteria floribunda* agglutinin, WFA), fibronectin (FN), and representatives of the cytoskeleton (β-actin; β3-tubulin; neurofilament light chain, NF-L; microtubule-associated protein 2, MAP2) in the neocortex of mice after 24 h of focal cerebral ischemia. Combinations of markers are compared between the non-affected **(A,C,E,G)** and the ischemia-affected hemisphere **(B-B”,D-D”’,F-F”’,H-H”’)**. Scale bars are labeled in related figures, while the scale bars in **(B”’,D”’,H”’)** are also valid for **(B’,B”,D’,D”,H’,H”)**, and the scale bar in **(F”’)** is also valid for **(F’,F”)**. Meanings of asterisk, arrows, and arrowheads are described in the main text.

The visualization of the vasculature was enabled by staining collagen IV as part of the basement membrane and lectin-based histochemistry with *Solanum tuberosum lectin* (STL) as a pan-vascular marker (Figures 3A,B). On the non-affected hemisphere, the fiber-like signal of MFAP5 appeared diffusely arranged without a clear association with the detected vasculature. In contrast, collagen IV immunoreactivity and STL staining were found to overlap partially (asterisks in Figure 3A). In the ischemic region, the signal of STL was primarily maintained, and that of collagen IV slightly increased. Still, the local arrangement with MFAP5 remained unchanged without an association between fibers and vascular structures (Figure 3B).

Constituents of the ECM were detected by the lectin *Wisteria floribunda* agglutinin (WFA) and by an antibody directed against fibronectin in combination with MFAP5 (Figures 3C,D). In the non-ischemic region, WFA showed perineuronal nets surrounding some neuronal cell bodies (asterisk in Figure 3C). However, simultaneous detection of WFA and MFAP5 in the same structure remained uncertain (arrow in Figure 3C). Fibronectin was observed mainly with a tube-like pattern probably related to the vasculature (arrowheads in Figure 3C) but without a regional association to MFAP5. On the ischemia-affected hemisphere, the immunosignal of MFAP5 appeared less concomitant than that originating from WFA staining toward the ischemic area, whereas fibronectin became gradually more visible. In line with the observations on the non-affected hemisphere, MFAP5 did not reveal an association with fibronectin in the ischemic region (Figure 3D).

### 3.3 Spatial relationships between MFAP5 and cytoskeletal elements

Cytoskeletal elements were visualized by immunohisto-chemical labeling of β-actin and NF-L as well as β3-tubulin and MAP2, each combined with MFAP5 (Figures 3E-H). In the non-ischemic hemisphere, β-actin mainly appeared as a diffuse signal exhibiting some accumulations partially arranged as surrounds of neuronal cell bodies (arrows in Figure 3E) but without an association with MFAP5. In contrast, NF-L was observed as filaments in neuronal processes (arrowheads in Figure 3E) and a surrounding formation of neuronal cell bodies (asterisk in Figure 3E), largely matching with the MFAP5 signal. On the ischemia-affected hemisphere, the filament-like structures that are immunopositive for NF-L and MFAP5 at the same time became markedly visible in the border zone (arrow in Figure 3F). Toward ischemia, the signals of NF-L and MFAP5 diminished and filaments appeared fragmented, whereas β-actin was diffusely maintained. Remarkably, the few remaining fragments of filaments in the ischemic region were predominantly double-labeled by NF-L and MFAP5 (arrowhead in Figure 3F).

In the non-affected hemisphere, β3-tubulin appeared regionally associated with the surface of neuronal cell bodies, especially with the initial part of some processes (arrows in Figure 3G), partially matching with MFAP5. MAP2 became visible in a surrounding formation concerning neuronal cell bodies and, more evident, as fibers with different diameters (arrowheads in Figure 3G) partially associated with axonal structures and MFAP5 (asterisk in Figure 3G). Comparable regional associations of MAP2, β3-tubulin, and MFAP5 were observed in the border zone on the ischemia-affected hemisphere. Toward ischemia, filaments detected by MAP2 and MFAP5 appeared fragmented, and the immunosignal of β3-tubulin was found markedly diffused (Figure 3H).

### 3.4 Morphological characteristics of MFAP5 and its regional association with NF-L and MAP2

**FIGURE 4:**
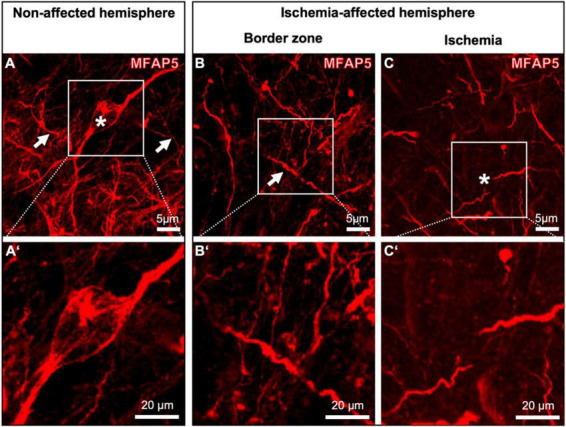
Single immunofluorescence labeling of microfibrillar-associated protein 5 (MFAP5). Representative micrographs of MFAP5-positive fibers at higher magnifications are compared between the non-affected **(A,A’)** and the ischemia-affected hemisphere, including the border zone **(B,B’)** and the ischemic region **(C,C’)**. Scale bars are labeled in related figures. Meanings of asterisks and arrows are described in the main text.

To further explore the local characteristics of MFAP5 under ischemic conditions, its staining patterns were compared between neocortical regions with higher magnification (Figures 4A-C’). In this approach, the non-affected hemisphere exhibited a dense network of MFAP5-positive fibers of different diameters (arrows in Figure 4A). Further, an accumulated MFAP5 signal was seen to surround some neuronal cell bodies and their processes (asterisk in Figure 4A). In the border zone of the ischemia-affected hemisphere, the MFAP5 signal also appeared as fibers but with morphological alterations in terms of a shrunken and partially twisted occurrence (arrow in Figure 4B). In the region that is mainly affected by ischemia, fibers visualized by MFAP5 appeared diminished and morphologically altered with a partially shrunken, fragmented, and twisted aspect (asterisk in Figure 4C).

**FIGURE 5:**
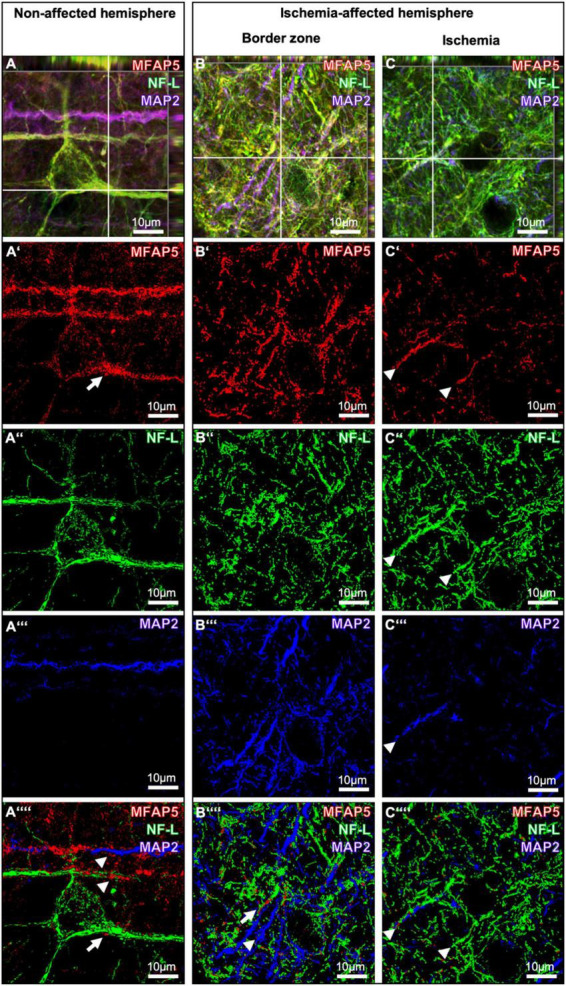
Triple immunofluorescence labeling of microfibrillar-associated protein 5 (MFAP5) neurofilament light chain (NF-L) and microtubule-associated protein 2 (MAP2) as representatives of the neuronal cytoskeleton. Confocal laser scanning microscopy of MFAP5, NF-L, and MAP2 in the non-affected **(A)** and the ischemia-affected hemisphere **(B,C)**. 3D surface reconstruction of z-stacked images from MFAP5, NF-L, and MAP in the non-affected **(A’-A””)** and the ischemia-affected hemisphere **(B’-B””, C’-C””)**. Scale bars are labeled in related figures. Meanings of arrows and arrowheads are described in the main text.

To explore regional associations of morphologically altered MFAP5-positive fibers and accumulations at cell bodies and their processes together with NF-L and MAP2, confocal microscopy (Figures 5A-C) was combined with 3D surface reconstruction of z-stacked images (Figures 5A’–C””). Thereby, MFAP5 partially matched with signals of MAP2 and NF-L on the non-affected hemisphere (arrowheads in Figure 5A””). Further, the accumulated MFAP5 signal with a surrounding and fiber-like appearance concerning neuronal cell bodies and their processes was observed to be closely associated with the NF-L signal (arrow in Figures 5A’,A””). In the border zone of the ischemia-affected hemisphere, a comparable pattern was found in a way that MFAP5 was closely associated with some filaments detected by NF-L and MAP2 (arrow and arrowhead in Figure 5B””). In the ischemic region, filament- or fiber-like structures appeared diminished, especially for MFAP5 and MAP2. At the same time, the close regional association of MFAP5 with NF-L or MAP2 was still apparent in some of the remaining filament or fiber fragments (arrowheads in Figure 5C””).

## 4 Discussion

The present study aimed to explore the local arrangement of MFAP5 with components of the NVU, ECM, and cytoskeleton in non- and ischemia-affected brain regions. This approach followed an earlier study that detected MFAP5 in the brain of mice with a mainly fiber-like pattern and a significantly reduced immunosignal due to experimental stroke (Höfling et al., 2023). MFAP5 has thus been discussed to represent an essential cell-stabilizing element that could be involved in the transition toward irreversible tissue damage during ischemia. Nonetheless, its regional characteristics have not yet been elucidated.

Using multiple immunofluorescence labeling, the present study confirmed the predominantly fiber-like structure of MFAP5 as described earlier (Höfling et al., 2023) and observed a partially surrounding formation concerning neuronal processes and cell bodies in neocortical areas. The region affected by focal cerebral ischemia exhibited an almost complete loss of markers typically visualizing neurons and a significantly diminished MFAP5 signal. Still, a few fiber-like structures were maintained with a thinned, partially fragmented, and twisted aspect.

Vascular and glial components of the NVU displayed no clear regional association with MFAP5. Regarding the ECM, MFAP5 was not regionally associated with fibronectin, and a relationship to perineuronal nets remained uncertain. Therefore, MFAP5 seems to have no direct association with the vasculature, related astrocytes, and the adjoining ECM in terms of the neurovascular matrix adhesion zone comprising elements such as collagen, integrins, and fibronectin (del Zoppo and Milner, 2006; Edwards and Bix, 2019; Michalski et al., 2020). Further, MFAP5 appears not to be associated with the endothelium and the adjacent proteoglycans, typically enabling interactions between the abluminal cytoskeleton and the intraluminal glycocalyx layer (Logsdon et al., 2021; Shi et al., 2025). However, the observed ischemic consequences for components of the NVU and ECM strengthened earlier studies also describing alterations of collagen IV as part of the vasculature (e.g., Hawkes et al., 2013; Michalski et al., 2020), MBP from oligodendrocytes (e.g., Michalski et al., 2018; Zuo et al., 2019), and fibronectin as constitute of the ECM (e.g., Summers et al., 2013; Michalski et al., 2020), as well as diminished perineuronal nets and morphologically altered microglia visualized by WFA and Iba1 (e.g., Härtig et al., 2022; Michalski et al., 2010) due to experimental stroke.

Among cytoskeletal elements, MFAP5 exhibited a local arrangement and morphological characteristics due to focal cerebral ischemia comparable to NF-L and MAP2. In detail, MFAP5, NF-L, and MAP2 immunosignals appeared predominantly as fibers or filaments and in a surrounding formation concerning neuronal processes and cell bodies. In the ischemic region, signals of all three markers considerably changed regarding intensity and morphological criteria. For NF-L and MAP2, the present findings are in line with earlier observations describing a gradually increased signal of NF-L at the ischemic border zone and a markedly diminished signal of MAP2 toward the ischemic region (Härtig et al., 2016; Mages et al., 2018; Mages et al., 2021). Notably, the present study yielded morphological changes in MFAP5 in terms of a thinned and partially twisted aspect due to ischemia, which has been described similarly for MAP2 (Härtig et al., 2016). Regarding other cytoskeletal elements investigated in the present study, i.e., β-actin and β3-tubulin, no clear regional association was observed for MFAP5. However, β3-tubulin provided a partial association in terms of an accumulating signal surrounding neuronal cell bodies. Therefore, MFAP5 seems to have more similarities with the larger-sized microtubules and neurofilaments that were described to be associated with neurons and especially their processes (Nahirney and Tremblay, 2021; Yuan et al., 2012).

Based on the observed similarities of MFAP5 with MAP2 and NF-L concerning morphological characteristics and the changes in the setting of experimental focal cerebral ischemia, MFAP5 is suggestive to represent an essential cytoskeletal element. Thus, comparable functional properties within the neuronal cytoskeleton can be discussed. Further, MFAP5 might be considered as a target for neuroprotective approaches to preserve neuronal integrity and inhibit the transition toward long-term tissue damage due to stroke. More basically, the present study provided robust evidence for using MFAP5 as an ischemia-sensitive marker that can be applied in preclinical studies dealing with pathophysiological issues in the setting of ischemia. As circulating levels of NF-L and MAP2 were found to correlate with clinical and tissue-related outcomes in stroke patients (e.g., Uphaus et al., 2019; Mages et al., 2021), MFAP5 could also be applied as a serum marker that may help to tailor individual treatments. At least for diseases other than stroke, notably different types of cancer and heart failure, studies were started to explore the predictive value of MFAPs regarding patients’ survival and outcome (Yang et al., 2017; Kujawa et al., 2022; Cheng et al., 2022).

The present study had a few limitations: First, while focusing on regional characteristics with NVU and ECM components, this work had a descriptive focus and thus cannot provide insight into the functional properties of MFAP5. Consequently, future studies are needed to explore the role of MFAP5 under ischemic conditions and within the group of cytoskeletal elements. In addition to the early stage, investigations might include later stroke phases with naturally different pathophysiological mechanisms. Second, the applied approach to visualize MFAP5 with cellular and non-cellular components of the NVU and ECM was solely based on multiple immunofluorescence labeling. This technique was chosen as it allows the visualization of various structures simultaneously, which is essential when exploring the local arrangement of different cellular and non-cellular components. Third, as previous work has demonstrated challenges when focusing on the direction of immunosignals, i.e., an increase or decrease was found to depend on the choice of primary antibodies and tissue preparation (Prehn et al., 2023), the present study did not include a quantification of respective signals. To ensure the comparability of brain slices used for descriptive analyses, the same pre-treatment and antibody composition were applied. However, regarding the observed similarities of MFAP5, MAP2, and NF-L, quantifying respected immunosignals and protein levels could help to confirm the observations made in this study and might thus be considered in further research.

## 5 Summary and outlook

This study provided a comprehensive description of the local arrangement of MFAP5 with components of NVU, ECM, and cytoskeleton in the setting of experimental stroke. While MFAP5 did not exhibit a clear regional association with glial cells, the vasculature, and parts of the ECM, it was observed in a fiber-like and partially surrounding formation associated with neuronal processes and cell bodies in neocortical areas. Thereby, MFAP5 displayed similarities with MAP2 and NF-L concerning morphological characteristics in non-affected brain regions and exhibited comparable changes due to ischemia. These observations suggest that MFAP5 represents an essential component of the neuronal cytoskeleton. Consequently, MFAP5 is of interest for future studies addressing its functional properties in ischemia and the potential for neuroprotective approaches.

## Data Availability

Data underlying this study will be made available upon reasonable request.
